# Establishing a comprehensive search strategy for Indigenous health literature reviews

**DOI:** 10.1186/s13643-021-01664-y

**Published:** 2021-04-19

**Authors:** Louise Harding, Caterina J. Marra, Judy Illes

**Affiliations:** Neuroethics Canada, Division of Neurology, Department of Medicine, University of British Columbia, 2211 Wesbrook Mall, Koerner S124, Vancouver, British Columbia V6T 2B5 Canada

**Keywords:** Methods, Systematic reviews, Scoping reviews, Academic databases, Search terms, Subject headings, Indigenous populations, Population health, Social justice, Ethics

## Abstract

**Background:**

Appropriate search strategies are essential to ensure the integrity and reproducibility of systematic and scoping reviews, as researchers seek to capture as many relevant resources as possible. In the case of Indigenous health reviews, researchers are met with the special challenge of creating a search strategy that can encompass this large, diverse population group with no universally agreed upon identification criteria.

**Main body:**

With an aim to promote improved review methodologies that uphold standards of justice, autonomy, and equity for Indigenous peoples and other heterogeneous populations, we describe critical gaps and approaches to close them. We report organizational and transparency issues around how Indigenous populations are indexed in several major databases, and draw on examples of published reviews and protocols to demonstrate the challenges inherent to creating a comprehensive search strategy.

**Conclusions:**

The conduct and communication of results from health literature research on global Indigenous populations are compromised by challenges of methodology that are rooted in the complexities inherent to defining Indigenous peoples. These challenges must be urgently addressed to improve this important field of inquiry moving forward.

**Supplementary Information:**

The online version contains supplementary material available at 10.1186/s13643-021-01664-y.

## Background

The integrity and reproducibility of systematic literature reviews can only be achieved if appropriate strategies exist to capture relevant resources and communicate results. This is an especially important challenge for researchers whose interests lie in health as it pertains to Indigenous communities and other populations for which there is a highly heterogeneous collection of literature. In this area of research, investigators may ask a broad array of pressing questions that, on the one hand, may be undifferentiated in some ways from studies that do not specifically have an Indigenous focus, such as incidence of disease in a community or region, health priorities, and perceptions about the risks and benefits of novel treatments. On the other hand, investigators may seek highly specific information from Indigenous communities as they pertain to perspectives, individual and community consent, data ownership and control, ways of knowing, and systemic inequities and disparities. The unique human rights challenges facing Indigenous peoples today, including the right to health and the use of their traditional territories, languages and cultures, make upholding standards of justice, autonomy and equity in systematic as well as scoping review research involving Indigenous peoples an imperative [1].

## Main text

Recently, we encountered critical gaps in a scoping review of the peer-reviewed literature about global Indigenous perspectives on brain health and wellness. The first significant one centers around the absence of a search strategy that effectively enables the comprehensive capture of relevant discourse surrounding Indigenous populations. No accepted universal definition of Indigenous exists to encompass the approximately 5000 distinct indigenous communities worldwide, comprising a population about 370 million people across 90 countries today nor do all communities view it as desirable, per se [2, 3]. Without such a definition—however broad or specific—researchers must generate their own working terms, rely on those created from an array of existing sources to formulate a search strategy, or some combination of creative approaches. The net effects of this gap are inadequate and inappropriate search strings for which efforts are not only inefficient, but fundamentally compromise the reproducibility of information captured and the authenticity of data interpretation.

Here, we provide examples and noteworthy attempts of how the task has been approached in the past, and point out accompanying shortfalls that pertain to a lack of transparency, inclusiveness, and acknowledgement of communities about which such research is conducted. We recognize that these are only select examples that illustrate our points. We make no value judgments and respect the intentions of the authors. We conclude with recommendations to improve the overall quality and scope of global Indigenous literature reviews.

### Indigenous database approach

Literature reviews depend on a selection of a representative set of databases from which researchers gather their resources. Examples of these are PubMed, Medline, the Cumulative Index to Nursing and Allied Health Literature (CINAHL), the Native Health Database, and Informit Indigenous Collections. In recent years, several research databases have been created that gather exclusively Indigenous content. The best available option is the large Informit Indigenous Collection, which is updated daily in consultation with Indigenous groups from both mainstream and obscure journals, and other sources [4]. However, we challenge whether their claim to provide content “of relevance to professionals and researchers involved in indigenous issues locally and globally” is accurate given that they only draw resources from limited regions of the world, namely, Australia, Indonesia, Malaysia, New Zealand, North America, and the Pacific. The collection is also limited with content that begins in 1977. An even more limited database by contrast—the Native Health database—archives work solely on North American Indigenous peoples [5]. While it does contain historical resources, it is functionally more limited than Informit Indigenous Collections, as it does not support the capability for complex searches or the export of articles.

Given such limitations, researchers must look to complement their literature searches with larger, non-specific databases. This generates a circular capture problem in that it necessarily involves the formulation of a string of search terms that encompass Indigenous populations globally; however, due to the ongoing challenges of creating a globally comprehensive search string, search returns frequently fail to support the intended purpose of the research.

### MeSH approach

The Medical Subject Headings (MeSH) indexing system created by the National Library of Medicine (NLM) for journal articles and books in the life sciences is used widely across major databases such as PubMed and Medline [6]. Other databases, such as the Cumulative Index to Nursing and Allied Health Literature (CINAHL), use an adapted MeSH set [7]. While they can be very useful for narrowing the results of a large literature search, when our team attempted to select MeSH terms for the search strategy, we encountered issues around consistency, validity, transparency, and organization.

In 2020, the National Library of Medicine (NLM) added the MeSH term “Indigenous Peoples” under their Ethnic Group category, defining its scope as: “Descendants who self-identify as members of a group who inhabited a country or region at the time when people of different cultures or ethnic origins arrived. They often maintain their distinct language, culture, and beliefs.” Meanwhile, the corresponding Indigenous peoples MeSH term in the Cumulative Index to Nursing and Allied Health Literature (CINAHL) is the “Native population of a country, region, or area.” Having two MeSH terms with the same name in different databases presents a challenge for researchers who may assume that the definition is consistent. However, settling on any single definition can be problematic in and of itself, as it may lack aspects critical in defining indigeneity for some groups, such as “acceptance by community” as per the Métis people of Canada [3]. Additionally, while the CINAHL MeSH term has multiple subheadings for a range of global Indigenous groups, the NLM system only includes a subheading for Alaska Natives without providing any explanation for why this specific group was singled out.

Neither database specifies how it systematically applies these very general definitions to the practical task of indexing articles. Transparency is not only important for the critical evaluation and use of subject headings but also in upholding accountability to the Indigenous experts who are presumably consulted in the creation of such definitions. An example where this lack of transparency causes a problem is that Roma peoples are indexed as a distinct ethnic group in the NLM MeSH system, but whether they are also classified as indigenous peoples, as some Roma people self-identify as both, is not indicated.

The NLM also has additional MeSH terms that encompass Indigenous groups under their Continental Population Groups heading, the result of which is a convoluted organizational scheme that can skew search results. The four ancestry groups are American Native Continental Ancestry Group, Oceanic Ancestry Group, African Continental Ancestry Group, and Asian Continental Ancestry Group. While the scope notes, entry terms, and related headings for the first two continental groups in this list only refer to Indigenous populations (e.g., Alaska Natives, Native Hawaiians), none of the information provided about the MeSH for African and Asian Ancestry Groups explicitly alludes to the concept of indigeneity. As such, many researchers will just include the American and Oceanic Ancestry Group MeSH in their search strategies, which may contribute to an overrepresentation of discourse about these Indigenous groups in global literature reviews.

### Key search terms approach

Keywords for Indigenous literature searches will typically, if not naturally, start with umbrella terms such as Indigenous, Aboriginal and Native. Using only these general search terms can be acceptable with adequate acknowledgement of their limitations, but many researchers pursue further breadth by adding a range of more specific terms. However, from what we have observed in the literature, this well-intentioned pursuit often results in the underrepresentation of Asian, European, and African Indigenous groups.

For example the authors of a systematic review about the factors that influence Indigenous peoples’ cancer treatment decision-making used two umbrella terms and three specific keywords referring to groups in the USA, Australia and New Zealand ([8] see Tranberg et al. 2016 in Additional file 1). As a result, the five articles that met their final inclusion criteria were about Australian Indigenous groups. We note the authors’ acknowledgement of their error in assuming that the search terms they chose could encompass all Indigenous groups globally. Similarly, in a systematic review comparing the incidence of suicide among Indigenous peoples with other populations the authors used a search list with 45 Indigenous communities for their review without justifying why those few communities were chosen to represent all Indigenous peoples ([9] see Pollock et al. 2018 in Additional file 1). While the authors acknowledge the possibility of bias given challenges around defining Indigenous peoples and their limited search terms they still assert that their search encompasses global Indigenous populations comprehensively.

Some authors have attempted to create their own complete lists of worldwide Indigenous communities with various successes and failings. For example, the systematic review protocol of Bishop-Williams et al. (2017) for studying the associations between weather parameters and acute respiratory infection outcomes in Indigenous and non-Indigenous peoples included an extensive list of community names compiled from two major international sources [10]. Theoretically, the scope of their list is global as it includes many international Indigenous groups. However, the search string is neither transparent nor complete; it includes small communities such as the Squamish and Haida Nations of the Pacific Northwest, but excludes other local nations such as Tsleil-Waututh and Cowichan (see Additional file 1). The authors acknowledge the challenges of their undertaking by stating that “[i]t is difficult to develop a search strategy that is robust enough to represent all nuances of the ter[m] Indigenous,” yet carry forth with the assumption that their search had an adequately global scope. The danger of such a dismissal is that the biases inherent in the original methods can be perpetuated if adopted uncritically by subsequent researchers. As a case in point, the authors of a 2020 scoping review examining how “global Indigenous mental health is impacted by meteorological, seasonal, and climatic changes” replicated the search strategy that was created by Bishop-Williams et al. (2017) with only very minor adjustments, and did not acknowledge any of the potential limitations or biases ([11], see Middleton et al., 2020 in Additional file 1). Such oversight can effectively silence the voices of certain indigenous communities and sustain a cycle of biased, incomplete, and inaccurate discourse, even while researchers endeavor to be productive advocates of justice for Indigenous peoples.

### Ways forward

Toward the goal of improving the rigor of methodology for global Indigenous literature reviews, we suggest four changes in current practice: establish one or more databases of literature about global Indigenous populations, improve transparency about classification strategies, create a living list of Indigenous communities, and promote critical thinking and reflection on the part of researchers to ensure the appropriateness and reproducibility of their search methods (Table 1). As all of these tasks will necessarily involve continued, deep collaboration with Indigenous Knowledge Holders, we also recommend that the work of these experts be identified and acknowledged.

**Table 1:** Challenges of global Indigenous search strategies and possible remedies

Methodological challenges	Remedies
Absence of a comprehensive Indigenous database.	Establishment of one or more databases that index global, historical, and contemporary literature about Indigenous populations.
Lack of transparency and accountability for how definitions of Indigenous peoples are operationalized.	Databases and other bodies that index Indigenous resources should provide explicit details about how they determine if a given community is classified as Indigenous under their definition.
Incomplete, piecemeal, and selective search term strings.	Creation of a list of global Indigenous communities.
Researchers erroneously assert that their search strategies encompass global Indigenous populations.	Researchers should critically consider and articulate the rationale for both the inclusion and exclusion of populations, and account for inherent limitations.

The establishment of a novel Indigenous database or improvement of an existing one such as Informit Indigenous Collections would functionally eliminate the need for researchers to create arduous and complex search strings to encompass global Indigenous populations. Such a database would allow researchers to seamlessly locate the relevant discourse regarding Indigenous peoples globally. As the many possible definitions of iIndigenous will need to be taken into account and applied transparently to indexing, multiple databases may be required to collectively serve this purpose.

Other databases and the organizations that index resources must also be transparent about how they are operationalizing their definitions of Indigenous peoples. For example, public access to the inclusion and exclusion criteria used by the National Library of Medicine for their MeSH terms would enable researchers to critically structure their search strategies.

Whether as part of this undertaking toward transparency or as a separate endeavor, the creation and maintenance of a comprehensive list of global Indigenous communities is needed. This will be resource-intensive to create, but its upkeep can be managed with a commitment to the task that is reflected in clearly defined operational procedures and a sustainable funding source. Such a list must skillfully incorporate a number of different possible definitions of Indigenous peoples, which will involve consulting with Indigenous communities about how they wish to be identified. Researchers may then use this list to create universally accepted working search strategies. Researchers may reference the date they accessed the search strategy in their methodology, and disclose any amendments they have made to answer their specific research questions. Such a list would also have an important impact on other types of work with Indigenous communities, such as serving advocacy and humanitarian purposes, and be a model for others.

Throughout the implementation of these three recommendations, consultation with Indigenous Knowledge Holders and other experts will be essential. Accordingly, we further recommend that the rationale for selecting these community contributors be noted explicitly, and their important work acknowledged.

Finally, until better methodologies for searching for global Indigenous groups are available, it is incumbent on researchers to critically consider and report which Indigenous groups and perspectives are most likely to be encompassed by the search strategies they formulate, and to acknowledge how this will impact their study results. Researchers who engage in this level of reflection at the time of developing their search protocol will likely find that using inclusive search terms and a broad range of databases will enhance the quality of their search. While out of scope for detailed discussion in this article, the inclusion of gray literature can also add breadth and an additional analytic layer. It is no longer acceptable for researchers to use biased or otherwise limited search strategies and then claim to capture global perspectives. In line with the concept of ethical reproducibility for the transparent reporting of research ethics methods used by biomedical researchers [12], literature reviewers also have an obligation to apply and report ethical considerations. Further, this interim effort will require support by international research bodies, major libraries, and academic publishers (e.g. Cochrane, National Library of Medicine, the American Association for the Advancement of Sciences) to set clear guidelines for search strategies including Indigenous populations.

## Conclusions

In our own endeavor to create a literature review search strategy that encompasses global Indigenous populations, we were not able to find a precedent study with methodology that satisfied the criteria of comprehensiveness, rigor, and transparency. This raised important concerns for us about the quality and representativeness of much global Indigenous literature review research, and disorganized or uncoordinated approaches, and one-off methodologies. The process of improvement has already slowly begun with the creation of Indigenous databases and improved subject headings, and we call for greater urgency and attention to the ethical imperative of moving away from the *status quo*^1^. Ultimately, until options are available to address these challenges, authors must take extra care to acknowledge the limitations of their search strategies in order to avoid perpetuating oppressive notions of who is Indigenous and who is not. Indeed, it is essential that the methodologies used in such research do not inadvertently perpetuate the very same oppressive paradigms they aim to remediate.

## Supplementary Information

**Additional file 1: Table A1.** Search terms used in four global Indigenous health literature reviews.

## Data Availability

Not applicable.

## Abbreviations

MeSH: Medical Subject Headings
NLM: National Library of Medicine
CINAHL: Cumulative Index to Nursing and Allied Health Literature

## Footnotes

1 Since writing the first version of this manuscript, the National Library of Medicine introduced its 2021 Medical Subject Headings (MeSH) that includes improved organization and categorization of North American Indigenous populations under the existing heading, Indians, North American.
