# Supplementary material for: Establishing a comprehensive search strategy for Indigenous health literature reviews
Source: Syst Rev. 2021 Apr 19;10:115. doi: 10.1186/s13643-021-01664-y (PMC8056629; doi:10.1186/s13643-021-01664-y)
Supplement: Supplementary file 1 — Additional file 1: Table A1. Search terms used in four global Indigenous health literature reviews. [file 13643_2021_1664_MOESM1_ESM.pdf]

Additional File 1

Towards a Comprehensive Search Strategy for Indigenous Literature Reviews

Louise Harding[1], Caterina J. Marra[1], Judy Illes[1]*.

[1]Neuroethics Canada, Division of Neurology, Department of Medicine, University of British Columbia, Vancouver, British Columbia, Canada.

*Correspondence to: jilles@mail.ubc.ca

This PDF file includes:

Table A1

**Authors and Publication Year**	**Search Terms Used**
Tranberg et al., 2016 (*8*)	Aboriginal OR Indigenous OR “Native Americans” OR Maori OR “Torres Strait Islander”
Pollock et al., 2018 (*9*)	"First Nation" Or "First Nations" Or "Pacific Islander" Or "Pacific Islanders" Or "Torres Strait Islander" Or "Torres Strait Islanders" Or Aborigin* Or Africa* Or Alaska* Or Aleut* Or Amerind* Or Arctic Or Aymara Or Bushmen Or Chukchi Or Chukotka* Or Circumpolar Or Eskimo* Or Greenland* Or Hmong Or Indian* Or Indigen* Or Inuit* Or Inupiaq Or Inupiat Or Khanty Or Maori* Or Mapuche Or Metis Or Native* Or Navaho* Or Navajo* Or Nenets Or Quechua Or Saami Or Sami Or Samoan* Or Siberia* Or Skolt Or Tribal Or Tribe* Or Xingu* Or Yup’ik Or Yupik Or Zuni
Bishop-Williams et al., 2017 (*10*)	Aasax OR Aboriginal OR “Aboriginal-Malay” OR Aborigine OR Achi OR Achuar OR Adibashi OR Adivasi OR Adivasis OR Afar OR Ainu OR Aka OR Akawai OR Akha OR Akie OR Akoula OR Akurio OR Akwoa OR “al-Kaabneh” OR “al-Asarmeh” OR “al-Ramadin” OR “al-Rshaida” OR “Alaska Native*” OR Aleut OR Alutor OR Amazigh OR Ambo OR “American Indian*” OR Ameridian OR Amuesha OR Anak OR “Andean Kichwe” OR Andoa OR Andorrans OR Angaite OR Anikhwe OR Anu OR Arara OR Arawak OR “Arawak-Taino” OR Arwak OR Ashaninka OR Atayal OR Austronesian OR “Ava Guarani” OR Awajun OR Awa OR Awakateco OR Aweer OR Ayeoreo OR Aymara OR Ayoreo OR Aztec OR Baaka OR Baantonu OR Babi OR Bahnar OR Babongo OR Bacwa OR Bagame OR Bagombe OR Bagyeli OR BaGyeli OR Bajuni OR Baka OR Bakgalagadi OR Bakola OR Bakongo OR Bakoya OR Balala OR Bambara OR Bambuti OR Bantu OR Barabaig OR Bariba OR Barimba OR Basarwa OR Bassari OR Batwa OR Bawarwa OR BaWka OR Bawn OR BaYeyi OR Bedzam OR Benet OR Berabis OR Berawan OR Berber OR Berbers OR Bidayuh OR Bigombe OR Biharis OR Bilma OR Bisayah OR Bobo OR Boeschs OR Bofi OR Boni OR Bonis OR Boranna OR Boro OR Bororo OR Boruka OR Botsarwa OR Bozo OR Brao OR Bribri OR “Bri Bri” OR Brunca OR Bugakhwe OR Bulu OR Bumipeuteras OR Bunak OR Bunun OR Bwiti OR Cabecar OR Cacaopera OR Campeche OR Carib OR Caribs OR “Ch’orti” OR Chachi OR Chaima OR Chakma OR Chalchiteco OR Chamorro OR Chamorru OR Chamorous OR “Chao-Khao” OR “Chao Ley” OR Charrua OR Chelkancy OR Chiapas OR Chibcha OR Chibchense OR Chipaya OR Chiriguano OR Chiquito OR Chiquitano OR Chorotega OR Chorti OR Cofan OR Chuaa OR Chuj OR Chukchi OR Chulymcy OR Chuvancy OR Ciboney OR “Ciboney-Taino-Arawak” OR “Cocama-Cocomilla” OR Colla OR Copts OR Cotier OR Cree OR Cumanagoto OR Dabou OR Dagiri OR Dahalo OR Danisi OR

(*Cont'd:* Bishop-Williams et al., 2017)	Daroobe OR Datoga OR Daza OR Degar OR Deti OR Diaguita OR Dinka OR Dioula OR Ditammari OR Dogon OR Dolgan OR Doma OR Dukha OR Dusun OR Ebrie OR "egga hodaabe" OR Elmolo OR "El Mono" OR Embera OR Emerillon OR Ency OR Endorois OR "Enlhet Norte" OR Enxet OR "Enxet Sur" OR Epera OR Eskimo OR "Ese Eja" OR Evenk OR Ewondo OR Fatukuku OR "First Nation*" OR "Forest dwell*" OR Fuegian OR Fulani OR Fulbe OR Galibia OR Galibi OR Garifuna OR Gaoshan OR Gio OR Guadalcanese OR Guana OR Guaicuru OR Guarani OR "Guarani Mbya" OR Guyami OR Guaymi OR Guerrero OR Gurani OR Guransi OR Gurung OR "G//ana" OR "G/wi" OR "Gwich'in" OR Hadzabe OR Hadza OR Haida OR Herero OR Hidalgo OR "Hill People" OR "Hill Person" OR Hmong OR Hoa OR Huambisa OR Huastec OR Hui OR Huetar OR Hutu OR Iban OR Igotot OR "Ik" OR Imazighn OR Imazighen OR Indigenous OR "Indigenous Palestinians" OR Ingarico OR Inuit OR Inupiat OR Inuvialut OR Iroquoian OR Itelmen OR "Itza'" OR Ixil OR Jacalteco OR "Jahalin Bedouin" OR Jarai OR Jivi OR Jumma OR "Ju'hoansi" OR "K'iche" OR "Ka Pei Aina" OR Kachin OR Kaiowa OR Kalanga OR Kalina OR "Kalina-go" OR Kalinago OR "Kalinago-Taino" OR "Kali'na" OR Kamchadal OR Kanak OR "Kanaka Maoli" OR Kanuri OR Kaqchikel OR Karamajong OR Karenni OR Kavalan OR Kayapo OR Kawashkar OR Kayan OR Kazakh OR Kedayan OR Kelait OR Kenyah OR Kereki OR Kety OR "Khali'nago" OR Khamu OR Khanty OR Khengs OR "Khmer Krom" OR Khoekhoe OR "Khoe-San" OR Khoikhoi OR Khoisan OR Khomani OR "Khudro Nrigoshthhi" OR Khumi OR Khwe OR Khyang OR Kichwas OR Kipsigis OR Kirdi OR Koba OR Koryak OR Krio OR Krohn OR Kua OR Kumandincy OR Kuna OR Kuy OR Kwisi OR Lahu OR Lao OR "Laotian Tribes" OR Lenca OR Lickanantay OR Limbu OR Lisu OR Livs OR Lobi OR Lokono OR Loma OR Lua OR Lumad OR "Lunda-Chokwe" OR Lushai OR Maasai OR Macourai OR Macuxi OR Macuzi OR Magar OR Makasae OR Makuxi OR Malagasy OR Malakote OR Malay OR "Malayo-Polynesian" OR Maleku OR Mangyan OR Mani OR Mano OR Mansi OR Maori OR Mam OR Manjo OR Marma OR "Marsh Dwellers" OR Mapuche OR Maskoy OR "masyarakat adat" OR Mataco OR "Mataco Matguayo" OR Matagulpa OR Maya OR "Maya Chorti" OR Mayagna OR Mbanderu OR Mbini OR Mbororo OR Mbukushu OR Mbundu OR Mbuti OR Mbri OR Mbya OR Mdendjele OR Melanesian OR "Melanesian-Papauan" OR Mestico OR Mestizo OR Merina OR Metis OR Miao OR Mien OR Mikaya OR Miskito OR Miskitu OR Misquito OR Mixte OR Mnong OR Mogeno OR "Mon-Khmer" OR Montagnards OR Mopan OR Moxeno OR Mozabite OR Mpukushu OR Mru OR Muong OR Murut OR "N/oakhwe" OR "N'guigmi" OR Nagas OR Nahoa OR Nahua OR Nahuatl OR Nama OR Nambiquara OR Nanaicy OR Nandeva OR "Nandevi Guarani" OR Naro OR "Naso Tjerdi" OR Native* OR "Native American*" OR "Native Hawai'ian*"

(*Cont'd:* Bishop-Williams et al., 2017)	OR Negidalcy OR Negeri OR Negrito OR Nemadi OR Nenets OR Nganasan OR Ngabe OR Ngobe OR "Ngobe-Bugle" OR Nivkhy OR Nuer OR "Nyaneka-Nkumbi" OR Oaxaca OR Ocanxiu OR Ogiek OR Ogoni OR Ojibway OR Okinawans OR "Orang Asli" OR Orochi OR Oroki OR Otomi OR Ovimibundu OR Oyampi OR "Pai Tavytera" OR Paiwan OR Palenqueros OR Palikur OR Pankho OR Patamona OR Pech OR Pemon OR Peul OR Peulh OR Penan OR Piaroa OR "Ping Pu" OR Pipil OR Pocomam OR Pokot OR Poqomam OR "Poqomchi'" OR Puebla OR Punan OR Puyuma OR "Q'anjob'al" OR "Q'eqchi" OR Qawasqar OR Qicaque OR Quechua OR Quenchua OR "Quintano Roo" OR Qom OR Rai OR Raisales OR Rakhine OR Rama OR Rapanui OR "Rapa Nui" OR Raute OR Rhade OR Roraima OR Rotumans OR Rukai OR Saami OR Sabaot OR Saharawis OR Saisyat OR Sakapulteco OR Sakizaya OR Sami OR (San AND Africa) OR Sanapan OR Sanapana OR Sandawe OR "Santa Rosa Carib" OR Sanya OR Saramancas OR ("Scheduled Tribes" AND India) OR Secoya OR Sediq OR Selkup OR Semang OR Sengwer OR Senoi OR Shan OR Sherpa OR Shipibo OR "Shipibo-Conibo" OR Shiwiar OR Shorcy OR Shua OR Shuar OR Siona OR Sipakapense OR Soioty OR "South Sea Islander*" OR Stieng OR "Sumu-Mayangna" OR Sutiaba OR Tachangya OR "Tai-Kadai" OR Taino OR "Taino-Kalingo" OR Tamang OR Tampuan OR Tapeba OR Tapebo OR Tareno OR Taurepang OR Tawahka OR Tazy OR Teda OR Teenek OR Teko OR Tektiteko OR Telengity OR Teleuty OR Temenbe OR Teribe OR Tesker OR Thakali OR Tharu OR Thao OR Tikuna OR Tikigaq OR Tirio OR Toba OR "Toba Maskoy" OR Tofolar OR Tolai OR Toloupan OR Tomarao OR Topnaars OR "Torres Straight Islander*" OR Totonac OR Toubou OR Truku OR Tsexakhwe OR Tripura OR Tsaatan OR Tsachila OR Tsou OR Tsumkwe OR Tshwa OR Tuareg OR Tuaregare OR Tubolar OR Tubu OR Tugen OR Tukano OR Tupi OR Tutong OR Tutsi OR "Tuvin-Todjin" OR Twa OR Tyua OR "Tz'utujil" OR Tzeltal OR Tzotzil OR Uchay OR Udege OR Ulchi OR "Ureueu-Wau-Wau" OR Uru OR Uspanteko OR Vadda OR Vadema OR Vai OR Veddhas OR Veps OR Vyadha OR "Waaniy-a-Laato" OR Waata OR Wadoma OR Wagashi OR Wapaichana OR Waorani OR Wapixana OR Warao OR Warrau OR "Warrau Wayana" OR Wayampi OR Wayana OR Wayeyi OR Wayuu OR Wichi OR Wodaabe OR Wounaan OR Xinka OR Yaaku OR Yami OR Yamana OR Yanomami OR Yukpa OR Yvytoso OR Zamuco OR Zapara OR Zapotec OR "!Xoo" OR "//'Xauesi" OR "/Xaisa" OR "'Akateco" OR ((Indigenous OR Aboriginal OR Native) AND (Ache OR Algonquin OR Amis OR Bedouin OR Bugle OR Bushmen OR Dakota OR Dan OR Fang OR Herder OR Herdsmen OR Indian* OR Karen OR Maroon OR Mohawk OR Mon OR pastoralist* OR Papua OR Pear OR Potters OR Pygmy OR Pygmy OR Rade OR Roma OR Sab OR Squamish OR Tay OR Trio OR Yucatan))

Middleton et al., 2020 (*11*)	Aasax OR Aboriginal OR "Aboriginal-Malay" OR Aborigine OR Achi OR Achuar OR Adibashi OR Adivasi OR Adivasis OR Afar OR Ainu OR Aka OR Akawai OR Akha OR Akie OR Akoula OR Akurio OR Akwoa OR "al-Kaabneh" OR "al-Asarmeh" OR "al-Ramadin" OR "al-Rshaida" OR "Alaska Native" OR "Alaska Natives" OR Aleut OR Alutor OR Amazigh OR Ambo OR "American Indian" OR "American Indians" OR Ameridian OR Amuesha OR Anak OR "Andean Kichwe" OR Andoa OR Andorrans OR Angaite OR Anikhwe OR Anu OR Arara OR Arawak OR "ArawakTaino" OR Arwak OR Ashaninka OR Atayal OR Austronesian OR "Ava Guarani" OR Awajun OR Awa OR Awakateco OR Aweer OR Ayeoreo OR Aymara OR Ayoreo OR Aztec OR Baaka OR Baantonu OR Babi OR Bahnar OR Babongo OR Bacwa OR Bagame OR Bagombe OR Bagyeli OR BaGyeli OR Bajuni OR Baka OR Bakgalagadi OR Bakola OR Bakongo OR Bakoya OR Balala OR Bambara OR Bambuti OR Bantu OR Barabaig OR Bariba OR Barimba OR Basarwa OR Bassari OR Batwa OR Bawarwa OR BaWka OR Bawn OR BaYeyi OR Bedzam OR Benet OR Berabis OR Berawan OR Berber OR Berbers OR Bidayuh OR Bigombe OR Biharis OR Bilma OR Bisayah OR Bobo OR Boeschs OR Bofi OR Boni OR Bonis OR Boranna OR Boro OR Bororo OR Boruka OR Botsarwa OR Bozo OR Brao OR Bribri OR "Bri Bri" OR Brunca OR Bugakhwe OR Bulu OR Bumipeuteras OR Bunak OR Bunun OR Bwiti OR Cabecar OR Cacaopera OR Campeche OR Carib OR Caribs OR "Ch'orti" OR Chachi OR Chaima OR Chakma OR Chalchiteco OR Chamorro OR Chamorru OR Chamorous OR "Chao-Khao" OR "Chao Ley" OR Charrua OR Chelkancy OR Chiapas OR Chibcha OR Chibchense OR Chipaya OR Chiriguano OR Chiquito OR Chiquitano OR Chorotega OR Chorti OR Cofan OR Chuaa OR Chuj OR Chukchi OR Chulymcy OR Chuvancy OR Ciboney OR "Ciboney-Taino-Arawak" OR "CocamaCocomilla" OR Colla OR Copts OR Cotier OR Cree OR Cumanagoto OR Dabou OR Dagiri OR Dahalo OR Danisi OR Daroobe OR Datoga OR Daza OR Degar OR Deti OR Diaguita OR Dinka OR Dioula OR Ditammari OR Dogon OR Dolgan OR Doma OR Dukha OR Dusun OR Ebrie OR "egga hodaabe" OR Elmolo OR "El Mono" OR Embera OR Emerillon OR Ency OR Endorois OR "Enlhet Norte" OR Enxet OR "Enxet Sur" OR Epera OR Eskimo OR "Ese Eja" OR Evenk OR Ewondo OR Fatukuku OR "First Nation" OR "First Nations" OR "Forest dweller" OR "Forest dwellers" OR Fuegian OR Fulani OR Fulbe OR Galibia OR Galibi OR Garifuna OR Gaoshan OR Gio OR Guadalcanese OR Guana OR Guaicuru OR Guarani OR "Guarani Mbya" OR Guyami OR Guaymi OR Guerrero OR Gurani OR Guransi OR Gurung OR "G//ana" OR "G/wi" OR "Gwich'in" OR Hadzabe OR Hadza OR Haida OR Herero OR Hidalgo OR "Hill People" OR "Hill Person" OR Hmong OR "Hoa" OR Huambisa OR Huastec OR Hui OR Huetar OR Hutu OR Iban OR Igotot OR "Ik" OR Imazighn OR Imazighen OR Indigenous OR "Indigenous Palestinians" OR Ingarico

(*Cont'd:* Middleton et al., 2020)	OR Inuit OR Inupiat OR Inuvialut OR Iroquoian OR Itelmen OR "Itza'" OR Ixil OR Jacalteco OR "Jahalin Bedouin" OR Jarai OR Jivi OR Jumma OR "Ju'hoansi" OR "K'iche" OR "Ka Pei Aina" OR Kachin OR Kaiowa OR Kalanga OR Kalina OR "Kalina-go" OR Kalinago OR "Kalinago-Taino" OR "Kali'na" OR Kamchadal OR Kanak OR "Kanaka Maoli" OR Kanuri OR Kaqchikel OR Karamajong OR Karenni OR Kavalan OR Kayapo OR Kawashkar OR Kayan OR Kazakh OR Kedayan OR Kelait OR Kenyah OR Kereki OR Kety OR "Khali'nago" OR Khamu OR Khanty OR Khengs OR "Khmer Krom" OR Khoekhoe OR "KhoeSan" OR Khoikhoi OR Khoisan OR Khomani OR "Khudro Nrigoshthhi" OR Khumi OR Khwe OR Khyang OR Kichwas OR Kipsigis OR Kirdi OR Koba OR Koryak OR Krio OR Krohn OR Kua OR Kumandincy OR Kuna OR Kuy OR Kwisi OR Lahu OR Lao OR "Laotian Tribes" OR Lenca OR Lickanantay OR Limbu OR Lisu OR Livs OR Lobi OR Lokono OR Loma OR Lua OR Lumad OR "Lunda-Chokwe" OR Lushai OR Maasai OR Macourai OR Macuxi OR Macuzi OR Magar OR Makasae OR Makuxi OR Malagasy OR Malakote OR Malay OR "Malayo-Polynesian" OR Maleku OR Mangyan OR Mani OR Mano OR Mansi OR Maori OR Mam OR Manjo OR Marma OR "Marsh Dwellers" OR Mapuche OR Maskoy OR "masyarakat adat" OR Mataco OR "Mataco Matguayo" OR Matagulpa OR Maya OR "Maya Chorti" OR Mayagna OR Mbanderu OR Mbini OR Mbororo OR Mbukushu OR Mbundu OR Mbuti OR Mbri OR Mbya OR Mdendjele OR Melanesian OR "Melanesian-Papauan" OR Mestico OR Mestizo OR Merina OR Metis OR Miao OR Mien OR Mikaya OR Miskito OR Miskitu OR Misquito OR Mixte OR Mnong OR Mogeno OR "MonKhmer" OR Montagnards OR Mopan OR Moxeno OR Mozabite OR Mpukushu OR Mru OR Muong OR Murut OR "N/oakhwe" OR "N'guigmi" OR Nagas OR "Nahoa" OR Nahua OR Nahuatl OR Nama OR Nambiquara OR Nanaicy OR Nandeva OR "Nandevi Guarani" OR Naro OR "Naso Tjerdi" OR Native* OR "Native American" OR "Native Americans" OR "Native Hawai'ian" OR "Native Hawai'ians" OR Negidalcy OR Negeri OR Negrito OR Nemadi OR Nenets OR Nganasan OR Ngabe OR Ngobe OR "Ngobe-Bugle" OR Nivkhy OR Nuer OR "Nyaneka-Nkumbi" OR Oaxaca OR Ocanxiu OR Ogiek OR Ogoni OR Ojibway OR Okinawans OR "Orang Asli" OR Orochi OR Oroki OR Otomi OR Ovimibundu OR Oyampi OR "Pai Tavytera" OR Paiwan OR Palenqueros OR Palikur OR Pankho OR Patamona OR Pech OR Pemon OR Peul OR Peulh OR Penan OR Piaroa OR "Ping Pu" OR Pipil OR Pocomam OR Pokot OR Poqomam OR "Poqomchi'" OR Puebla OR Punan OR Puyuma OR "Q'anjob'al" OR "Q'eqchi" OR Qawasqar OR Qicaque OR Quechua OR Quenchua OR "Quintano Roo" OR Qom OR Rai OR Raisales OR Rakhine OR Rama OR Rapanui OR "Rapa Nui" OR Raute OR Rhade OR Roraima OR Rotumans OR Rukai OR Saami OR Sabaot OR Saharawis OR Saisyat OR Sakapulteco OR Sakizaya OR Sami OR (San AND Africa) OR Sanapan OR Sanapana OR Sandawe

(*Cont'd:* Middleton et al., 2020)	OR "Santa Rosa Carib" OR Sanya OR Saramancas OR ("Scheduled Tribes" AND India) OR Secoya OR Sediq OR Selkup OR Semang OR Sengwer OR Senoi OR Shan OR "Sherpa" OR Shipibo OR "Shipibo-Conibo" OR Shiwiar OR Shorcy OR Shua OR Shuar OR Siona OR Sipakapense OR Soioty OR "South Sea Islander" OR "South Sea Islanders" OR Stieng OR "Sumu-Mayangna" OR Sutiaba OR Tachangya OR "Tai-Kadai" OR Taino OR "Taino-Kalingo" OR Tamang OR Tampuan OR Tapeba OR Tapebo OR Tareno OR Taurepang OR Tawahka OR Tazy OR Teda OR Teenek OR Teko OR Tektiteko OR Telengity OR Teleuty OR Temenbe OR Teribe OR Tesker OR Thakali OR Tharu OR "Thao" OR Tikuna OR Tikigaq OR Tirio OR Toba OR "Toba Maskoy" OR Tofolar OR Tolai OR Toloupan OR Tomarao OR Topnaars OR "Torres Straight Islander" OR "Torres Straight Islanders" OR Totonac OR Toubou OR Truku OR Tsexakhwe OR Tripura OR Tsaatan OR Tsachila OR Tsou OR Tsumkwe OR Tshwa OR Tuareg OR Tuaregare OR Tubolar OR Tubu OR Tugen OR Tukano OR Tupi OR Tutong OR Tutsi OR "Tuvin-Todjin" OR Twa OR Tyua OR "Tz'utujil" OR Tzeltal OR Tzotzil OR Uchay OR Udege OR Ulchi OR "Ureueu-Wau-Wau" OR Uru OR Uspanteko OR Vadda OR Vadema OR Vai OR Veddhas OR Veps OR Vyadha OR "Waaniy-a-Laato" OR Waata OR Wadoma OR Wagashi OR Wapaichana OR Waorani OR Wapixana OR Warao OR Warrau OR "Warrau Wayana" OR Wayampi OR Wayana OR Wayeyi OR Wayuu OR Wichi OR Wodaabe OR Wounaan OR Xinka OR Yaaku OR Yami OR Yamana OR Yanomami OR Yukpa OR Yvytoso OR Zamuco OR Zapara OR Zapotec OR "!Xoo" OR "//'Xauesi" OR "/Xaisa" OR "'Akateco" OR ((Indigenous OR Aboriginal OR Native) AND ("Ache" OR Algonquin OR Amis OR Bedouin OR "Bugle" OR Bushmen OR Dakota OR Dan OR Fang OR Herder OR Herdsmen OR Indian* OR Karen OR Maroon OR Mohawk OR Mon OR pastoralist* OR Papua OR "Pear" OR Potters OR Pygmy OR Pygymy OR Rade OR Roma OR Sab OR Squamish OR Tay OR Trio OR Yucatan))

Table A1. Search terms used in four global Indigenous health literature reviews.
